# Prognostic biomarkers related to breast cancer recurrence identified based on Logit model analysis

**DOI:** 10.1186/s12957-020-02026-z

**Published:** 2020-09-25

**Authors:** Xiaoying Zhou, Chuanguang Xiao, Tong Han, Shusheng Qiu, Meng Wang, Jun Chu, Weike Sun, Liang Li, Lili Lin

**Affiliations:** 1 Department of Nursing, Wuxi Higher Health Vocational Technology School, Wuxi, 2140128 Jiangsu China; 2 Department of Breast Thyroid Surgery, Central Hospital of Zibo, Zibo, 255036 Shandong China; 3 Department of Rehabilitation Medicine, Xinxiang Medical University, Xinxiang, 453000 Henan China; 4 Department of Pharmacy, Wuxi Higher Health Vocational Technology School, No. 305, Xinguang Road, Wuxi, 214028 Jiangsu China

**Keywords:** Breast cancer, Prognosis, Recurrence, Logit regression model

## Abstract

**Background:**

This study intended to determine important genes related to the prognosis and recurrence of breast cancer.

**Methods:**

Gene expression data of breast cancer patients were downloaded from TCGA database. Breast cancer samples with recurrence and death were defined as poor disease-free survival (DFS) group, while samples without recurrence and survival beyond 5 years were defined as better DFS group. Another gene expression profile dataset (GSE45725) of breast cancer was downloaded as the validation data. Differentially expressed genes (DEGs) were screened between better and poor DFS groups, which were then performed function enrichment analysis. The DEGs that were enriched in the GO function and KEGG signaling pathway were selected for cox regression analysis and Logit regression (LR) model analysis. Finally, correlation analysis between LR model classification and survival prognosis was analyzed.

**Results:**

Based on the breast cancer gene expression profile data in TCGA, 540 DEGs were screened between better DFS and poor DFS groups, including 177 downregulated and 363 upregulated DEGs. A total of 283 DEGs were involved in all GO functions and KEGG signaling pathways. Through LR model screening, 10 important feature DEGs were identified and validated, among which, *ABCA3*, *CCL22*, *FOXJ1*, *IL1RN*, *KCNIP3*, *MAP2K6*, and *MRPL13*, were significantly expressed in both groups in the two data sets. *ABCA3*, *CCL22*, *FOXJ1*, *IL1RN*, and *MAP2K6* were good prognostic factors, while *KCNIP3* and *MRPL13* were poor prognostic factors.

**Conclusion:**

*ABCA3*, *CCL22*, *FOXJ1*, *IL1RN*, and *MAP2K6* may serve as good prognostic factors, while *KCNIP3* and *MRPL13* may be poor prognostic factors for the prognosis of breast cancer.

## Highlights

1. LR model screened 10 important feature DEGs.
2. *ABCA3*, *CCL22*, *FOXJ1*, and *IL1RN* were good prognostic factors.
3. *KCNIP3* and *MRPL13* were poor prognostic factors.

## Background

Breast cancer is one of the most frequent malignancies in both developed and developing countries, with an estimated 1.5 million new cases per year [1]. Although mammography screening and biomarker testing can improve early diagnosis, tumor recurrence, local, or distant metastases, following conventional therapies is a leading cause of morbidity and mortality in breast cancer patients [2, 3]. As a result, it is critical to identify biomarkers that could predict the recurrence or disease-free survival (DFS) of breast cancer.

Traditionally, the most widely used prognostic factors for the recurrence of breast cancer included tumor size, histologic grade, and the number of axillary lymph nodes with metastasis [4, 5]. These prognostic factors can supply independent prognostic information for patients with breast cancer, whereas they are not suitable for optimal patient management, especially as we move towards the era of personalized treatment [6]. A recent study has suggested that a variety of gene expression changes have occurred in early or precancerous breast cancer, which often precede the appearance of clinical symptoms and can serve as molecular biomarkers of early breast cancer [7]. Thus, a great deal of researches has been devoted to the development and validation of molecular biomarkers that cannot only provide prognostic information but also predict the response to therapy [8, 9]. Currently, many prognostic biomarkers for recurrence of breast cancer have been established, such as 21-gene Oncotype DX assay panel, 70-gene MammaPrit panel, 36-gene signature, PAM50-based Prosigna risk of recurrence (ROR) (NanoString), Breast Cancer Index (BCI) (bioTheranostics), and EndoPredict (EPclin) (Myriad Genetics) [10–12]. Although the findings above, our understanding of the molecular mechanisms of breast cancer recurrence is far from clear because of the molecular heterogeneity of breast cancer.

In this study, we aimed to further determine important genes related to the prognosis and recurrence of breast cancer by analyzing the breast cancer gene expression profile in TCGA database based on Logit regression (LR) model analysis and survival analysis. The results may help to provide more powerful biomarkers for the prognosis and recurrence of breast cancer.

## Materials and methods

### Data sources

Illumina HiSeq 2000 gene expression test data of breast cancer patients were downloaded from TCGA database (https://gdc-portal.nci.nih.gov/), involving a total of 1217 samples. After corresponding to the provided clinical information of the samples, the prognostic grouping was conducted according to the following rules [13]: breast cancer samples with recurrence and death were defined as poor DFS group, while samples without recurrence and survival beyond 5 years were defined as better DFS group. Finally, there were respectively 52 samples and 181 samples in poor and better DFS groups. Besides, after removing the samples without clinical information on breast cancer subtypes (Supplementary materials-table 1), 188 samples were left. Based on the subtypes of breast cancer, these samples in TCGA database were divided into four groups, including Basal (45 samples), Her2 (13 samples), LumA (94 samples), and LumB (36 samples). In addition, another gene expression profile dataset (GSE45725 [14]) of breast cancer was downloaded, which included 340 breast cancer tumor samples. The detection platform was GPL6883 Illumina humanref-8 v3.0 expression beadchip. After removing the samples that had not been followed-up for 3 years, the remaining samples were divided into DFS group (107 samples) and poor DFS group (20 samples), based on the 3-year survival associated with the clinical information. This dataset was used as the validation data. The histopathological data from TCGA and GSE45725 were shown in Supplementary materials-table 1 and Supplementary materials-table 2, respectively.

### Data preprocessing and differential expression analysis

After downloading the original expression level data, the *Z*-score transformation method [15, 16] was used to normalize the original data. Then, according to the grouping, and the R3.4.1 limma package version 3.34.7 [17] (https://bioconductor.org/packages/release/bioc/html/limma.html) was adopted for differentially expressed gene (DEG) screening for the better DFS and poor DFS group samples. False discovery rate (FDR) < 0.05 and |log_2_ fold change (FC)| > 1 were selected as the threshold for screening the DEGs. To verify whether DEGs can be used to distinguish samples with different prognostic conditions, bidirectional hierarchical clustering was conducted by the R3.4.1 pheatmap package version 1.0 [18]. (https://cran.r-project.org/web/packages/pheatmap/index.html) based on the Pearson correlation algorithm [19].

### Function enrichment analysis

Gene Ontology (GO) function (biological process (BP), molecular function (MF), and cellular component (CC)) and Kyoto Encyclopedia of Genes and Genomes (KEGG) [20] pathway annotation for DEGs was performed using DAVID version 6.8 [21, 22] (https://david.ncifcrf.gov/). *P* value less than 0.05 was selected as the threshold of enrichment significance. The DEGs that were enriched in the GO function and KEGG signaling pathway were selected for further analysis.

### Independent prognostic DEG screening

Based on the breast cancer tumor samples and the clinical prognostic information in TCGA dataset, the DEGs enriched in the GO function and KEGG signaling pathway were subjected to screening of significant prognostic correlation using the univariate cox regression analysis in R3.4.1 survival pack version 2.41-1 [23] (http://bioconductor.org/packages/survivalr/). Then, multivariate cox regression analysis was used to further screen independent prognostic DEGs, and log-rank *p* value less than 0.05 was selected as the threshold of significant correlation.

### LR model analysis

Based on the obtained independent prognostic DEGs, we used the glm function in R3.4.1 language to conduct LR model to screen important feature DEGs and classify the two groups of patients with different prognosis. All the genes with *p* < 0.05 were considered as important feature genes, and then, the accuracy was calculated based on the significant feature genes. Based on the expression characteristics of feature DEGs, all samples were divided into better and poor DFS groups in TCGA training dataset and GSE45725 validation dataset, respectively.

### Correlation analysis between LR model classification and survival prognosis

Based on the classification result of LR classification model in the training set and validation set, the Kaplan-Meier (KM) curve method in R3.4.1 survival package version 2.41-1 [15] was used to evaluate the correlation between the grouping conditions (better and poor DFS) and survival prognostic information. Then, the expression levels of important feature DEGs in TCGA training dataset and GSE45725 validation dataset were displayed. In addition, receiver operating characteristic (ROC) curve was drawn to compare the sensitivity and specificity. The area under the curve (AUC) was calculated from the ROC curve. The genes based on the LR models were also verified in the different subtypes of breast cancer (Basal, Her2, LumA, and LumB types).

## Results

### DEG screening

**Fig. 1:**
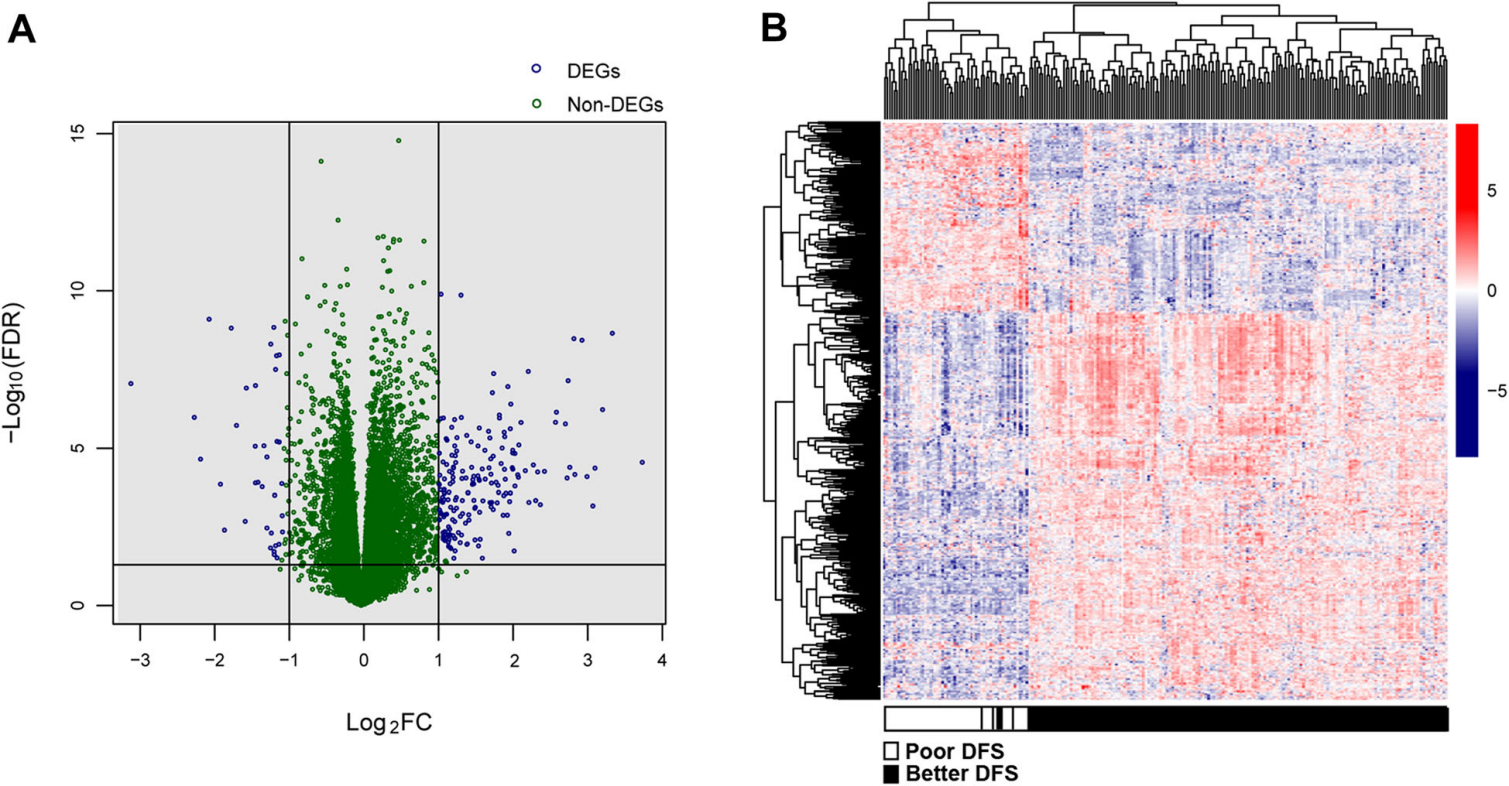
**a** The volcano plot of differentially expressed genes (DEGs). Blue circle represents DEGs, black horizontal line represents FDR < 0.05, and two black vertical lines represent |log_2_FC| > 1. **b** Bidirectional hierarchical clustering heatmap based on DEG expression level. White and black bars represent poor and better disease-free survival (DFS) breast cancer tumor samples, respectively

According to the DEG screening threshold (FDR < 0.05 and |log_2_FC| > 1), a total of 540 DEGs (better DFS vs. poor DFS) were screened, including 177 significantly downregulated and 363 significantly upregulated DEGs (Fig. 1a). The bidirectional hierarchical clustering heatmap based on DEG expression level is shown in Fig. 1b. It can be clearly seen from the figure that samples with similar gene expression patterns were stratified and clustered into the same group, indicating that the selected DEGs can well distinguish samples of different prognostic types.

### Function enrichment analysis

The downregulated and upregulated DEGs were respectively enriched into 26 and 89 GO terms as well as 6 and 7 KEGG signaling pathways, as shown in Table 1. The results showed that the significantly upregulated DEGs in the better DFS group were significantly enriched in BP functions such as immune response and defense response, CC terms associated with plasma membrane part, and MF terms of cytokine binding, and serine-type peptidase activity. Additionally, they were significantly involved in KEGG signaling pathways such as cell adhesion molecules, antigen processing and presentation, and chemokine signaling pathway. The downregulated DEGs were significantly related to the development of primary sexual characteristics, negative regulation of signal transduction, synapse part, and calcium-dependent protein binding and were involved in KEGG signaling pathways such as cell adhesion molecules, mTOR signaling pathway, and RNA degradation. A total of 283 DEGs were involved in all GO functions and KEGG signaling pathways.

**Table 1:** GO function node list (top 3) significantly enriched by upregulated and downregulated DEGs

	Category	Term	Count	***p*** value
Upregulation	BP	GO:0006955~immune response	67	1.270E−27
		GO:0006952~defense response	45	1.130E−13
		GO:0019882~antigen processing and presentation	14	9.100E−09
	CC	GO:0044459~plasma membrane part	75	9.160E−08
		GO:0005887~integral to plasma membrane	45	6.820E−06
		GO:0005886~plasma membrane	103	7.390E−06
	MF	GO:0019955~cytokine binding	10	2.910E−04
		GO:0008236~serine-type peptidase activity	10	8.588E−03
		GO:0017171~serine hydrolase activity	10	9.209E−03
	KEGG	hsa04514:Cell adhesion molecules (CAMs)	15	9.070E−06
		hsa04612:Antigen processing and presentation	12	1.040E−05
		hsa04062:Chemokine signaling pathway	17	3.180E−05
Downregulation	BP	GO:0045137~development of primary sexual characteristics	5	8.024E−03
		GO:0009968~negative regulation of signal transduction	6	1.340E−02
		GO:0007548~sex differentiation	5	1.409E−02
	CC	GO:0044456~synapse part	7	1.228E−02
		GO:0045202~synapse	8	2.139E−02
		GO:0005761~mitochondrial ribosome	3	3.516E−02
	MF	GO:0048306~calcium-dependent protein binding	3	1.372E−02
		GO:0005509~calcium ion binding	14	1.696E−02
		GO:0008083~growth factor activity	5	2.078E−02
	KEGG	hsa04514:Cell adhesion molecules (CAMs)	3	2.108E−02
		hsa04150:mTOR signaling pathway	2	2.884E−02
		hsa03018:RNA degradation	2	3.115E−02

### Independent prognostic DEG screening

Based on the 283 DEGs involved in all GO functions and KEGG pathways, a total of 186 prognostic DEGs were identified after univariate cox regression analysis by a combination of the 233 breast cancer tumor samples. Further multivariate cox regression analysis of the 186 prognostic DEGs screened 42 independent prognostic DEGs.

### LR model analysis

**Fig. 2:**
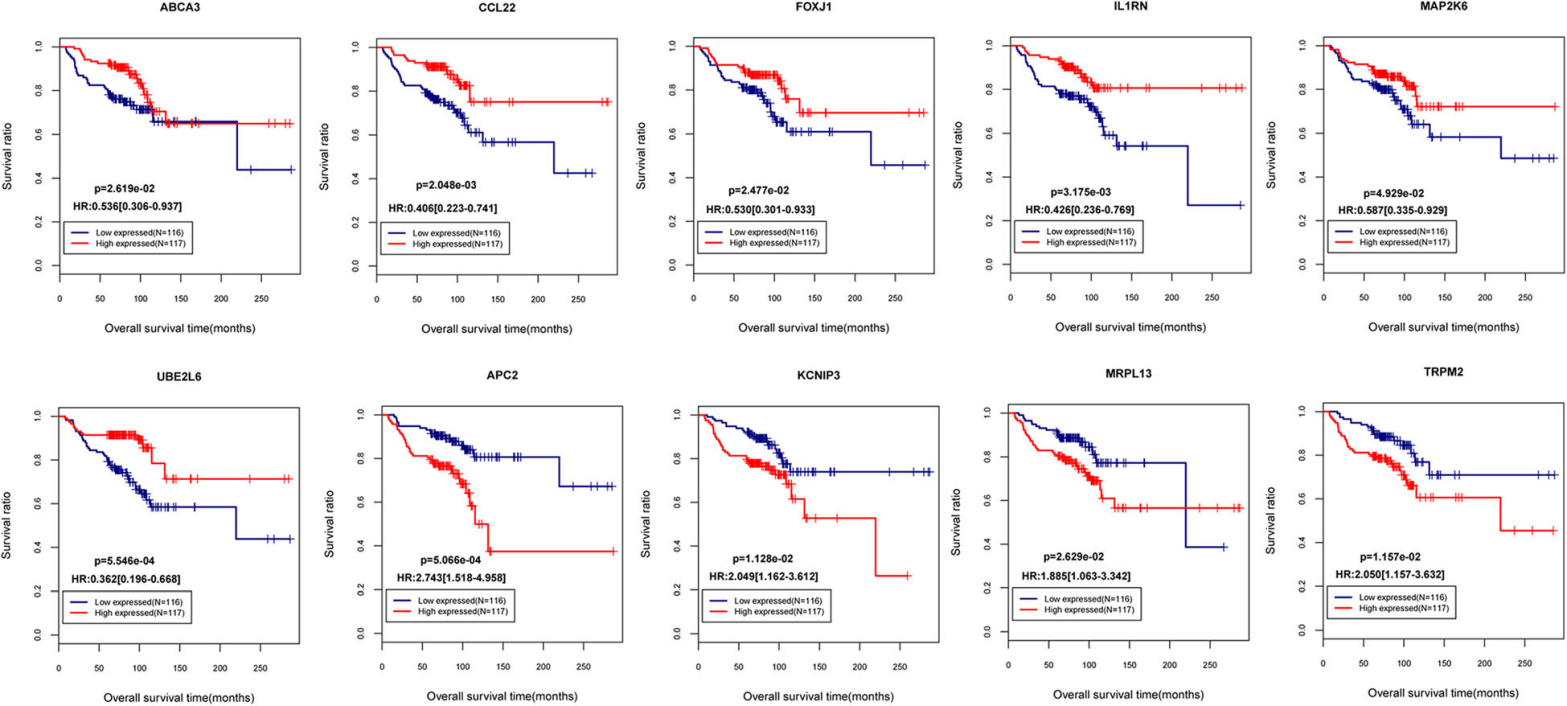
The KM curves of 10 important characteristic DEGs. Red represents the high-expression sample group, and blue represents the low-expression sample group

For the 42 DEGs that were significantly correlated with independent prognosis, LR model was used to screen important feature DEGs, and a total of 10 important feature DEGs were screened, as shown in Table 2. According to the median value of each DEG expression level, the training set samples were divided into high expression (expression level higher than the median value) and low expression (expression level lower than the median value), and the correlation between the samples in different expression level groups and survival prognosis was evaluated by KM curve method. As shown in Fig. 2, the *p* values of TP binding cassette subfamily a member 3 (*ABCA3*), C-C motif chemokine ligand 22 (*CCL22*), forkhead box J1 (*FOXJ1*), interleukin 1 receptor antagonist (*IL1RN*), mitogen-activated protein kinase kinase 6 (*MAP2K6*), ubiquitin conjugating enzyme E2 L6 (*UBE2L6*), APC regulator of Wnt signaling pathway 2 (*APC2*), potassium voltage-gated channel interacting protein 3 (*KCNIP3*), mitochondrial ribosomal protein L13 (*MRPL13*), transient receptor potential cation channel subfamily M member 2 (*TRPM2*) between high expression, and low expression were respectively 2.619E−02, 2.048E−03, 2.477E−02, 3.175E−03, 4.929E−02, 5.546E−04, 5.066E−04, 1.128E−02, 2.629E−02, and 1.157E−02, which indicated that these genes can be well distinguished. Based on the hazard ratio (HR) of these genes, *ABCA3* (0.536), *CCL22* (0.406), *FOXJ1* (0.530), *IL1RN* (0.426), *MAP2K6* (0.587), and *UBE2L6* (0.362) were good prognostic factors, which were significantly upregulated in the better DFS group. In contrast, *APC2* (2.743), *KCNIP3* (2.049), *MRPL13* (1.885), and *TRPM2* (2.050) were poor prognostic factors that were significantly upregulated in the poor DFS group. Based on the DEG expression level of 10 important feature genes, the LR model was used to classify the prognostic types of TCGA training set and GSE45725 validation data samples, respectively. In the GSE45725 validation dataset, 127 breast cancer samples were selected, of which 20 were breast cancer samples with recurrence within 3 years, and were defined as poor DFS group; 107 were samples with no recurrence and survival within 3 years and were defined as better DFS group. The fuzzy matrix of classification results is shown in Table 3.

**Table 2:** The important prognostic feature DEGs screened by Logit model

ID	B	SE	Df	***p*** value
UBE2L6	− 2.2813	0.7054	1	0.00122
ABCA3	− 1.5546	0.5185	1	0.00272
MAP2K6	− 0.7117	0.3443	1	0.03877
IL1RN	− 0.5372	0.2485	1	0.03064
FOXJ1	− 0.2375	0.0955	1	0.01285
CCL22	− 0.5239	0.2706	1	0.04529
APC2	0.6777	0.3478	1	0.04513
TRPM2	0.9379	0.3747	1	0.01232
KCNIP3	0.9632	0.3339	1	0.00392
MRPL13	3.0732	0.7985	1	0.00012

Note:*B* regression coefficient, *SE* Standard error, *Df* Degree of freedom

**Table 3:** The classification result in TCGA dataset and GSE45725 microarray dataset

TCGA	Predict			
	Class	Better DFS	Poor DFS	Percent (%)
Observed	Better DFS	172	9	95.03
	Poor DFS	14	38	73.08
Overall percent (%)				90.13
GSE45725	Better DFS	100	7	93.46
Observed	Poor DFS	6	14	70.00
Overall percent (%)				89.76

*DFS* Disease-free survival

### Correlation analysis between LR model classification and survival prognosis

**Fig. 3:**
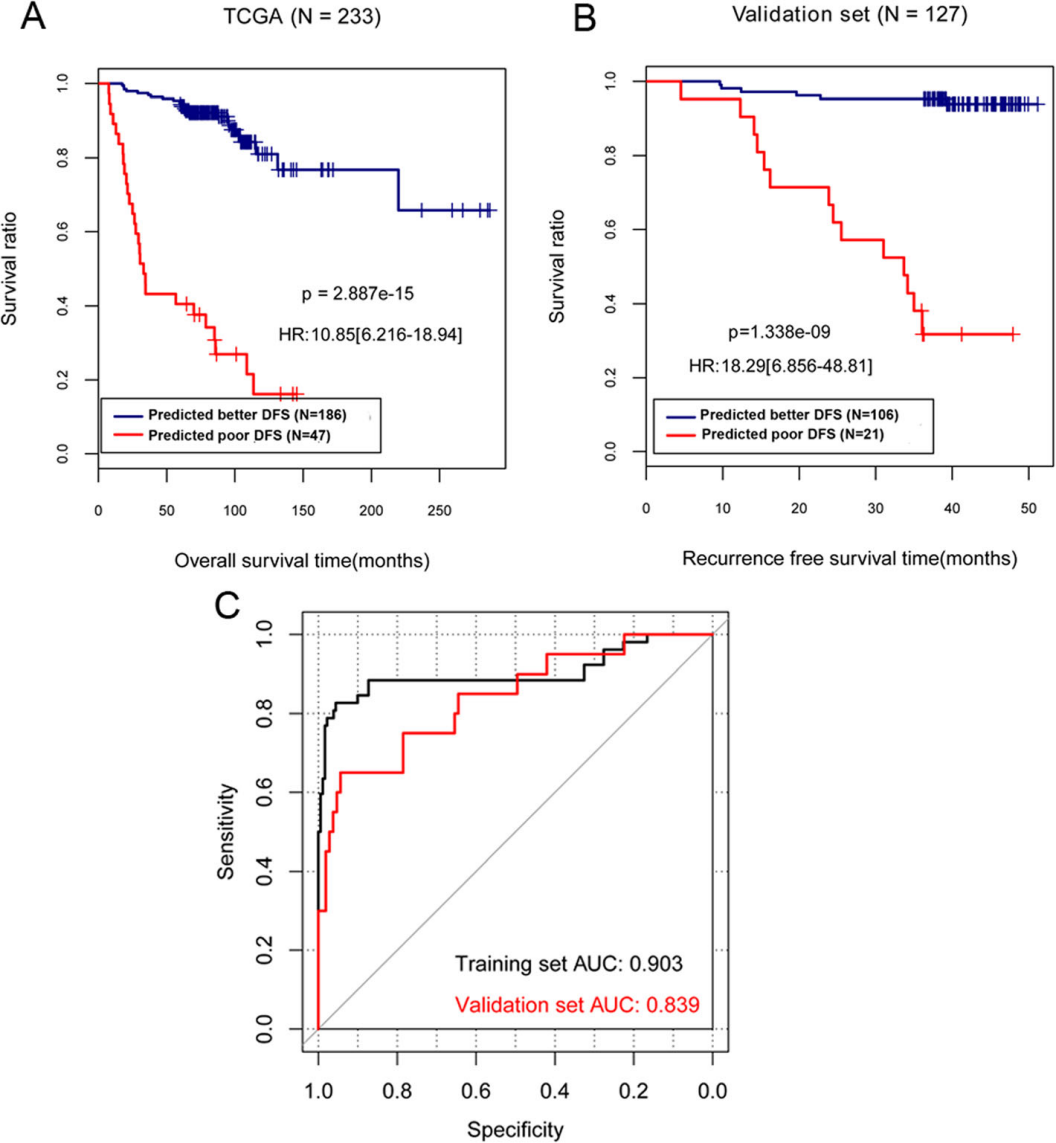
The KM curves of the correlation between the classification groups in TCGA training set (**a**) and GSE45725 validation set (**b**) and the actual survival prognosis based on the LR classification model. The blue and red curves represent the groups of good and poor prognosis samples predicted by the LR classification model, respectively. **c** Area under the curve (AUC) was calculated from the receiver operating characteristic (ROC) curve. Black and red curves represent the TCGA training set and GSE45725 validation set

There was a significant correlation between the grouping of samples after classification and the actual survival prognosis based on 10 important LR classification models (Fig. 3a, b). In addition, the AUC of the ROC curve in the training set and validation set were 0.903 and 0.839, respectively. These results demonstrated that the selected feature genes based on LR model can be well used to predict the prognosis and recurrence of breast cancer.

**Fig. 4:**
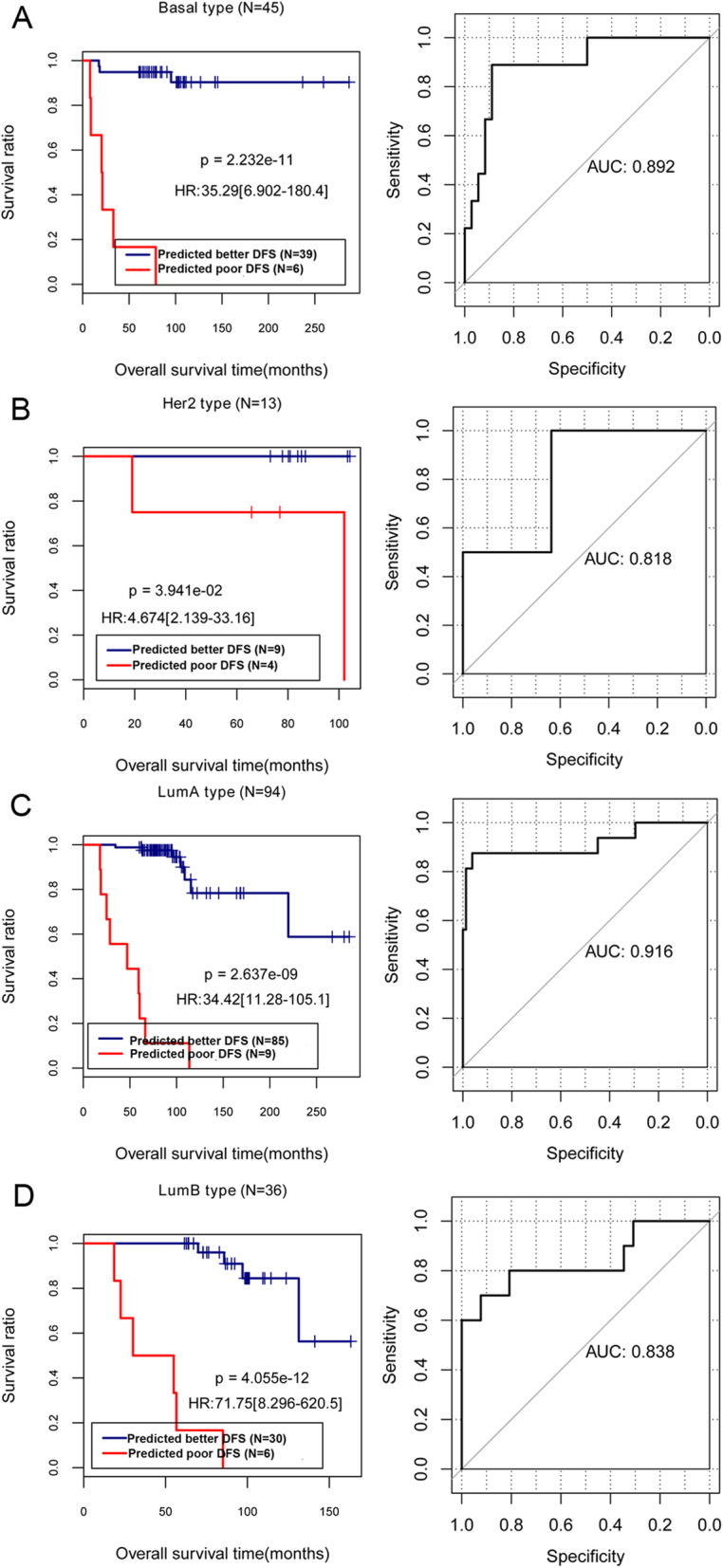
The KM curves and AUC were analyzed in the basal type (**a**), Her2 type (**b**), LumA type (**c**), and LumaB type (**d**) of breast cancer using TCGA dataset. The blue and red curves represent the groups of better and poor DFS samples predicted by the LR classification model, respectively

In the different subtypes of breast cancer, including basal type (Fig. 4a), Her2 type (Fig. 4b), LumA type (Fig. 4c), and LumB type (Fig. 4d), the prognosis of breast cancer predicted by the LR classification models was similar with the actual survival prognosis. Additionally, the AUCs of ROC curve in the basal type (Fig. 4a), Her2 type (Fig. 4b), LumA type (Fig. 4c), and LumB type (Fig. 4d) were 0.892, 0.818, 0.916, and 0.838, respectively. These results implied that the 10 important genes screened based on LR model can also be well utilized to predict the prognosis of different subtypes of breast cancer.

**Fig. 5:**
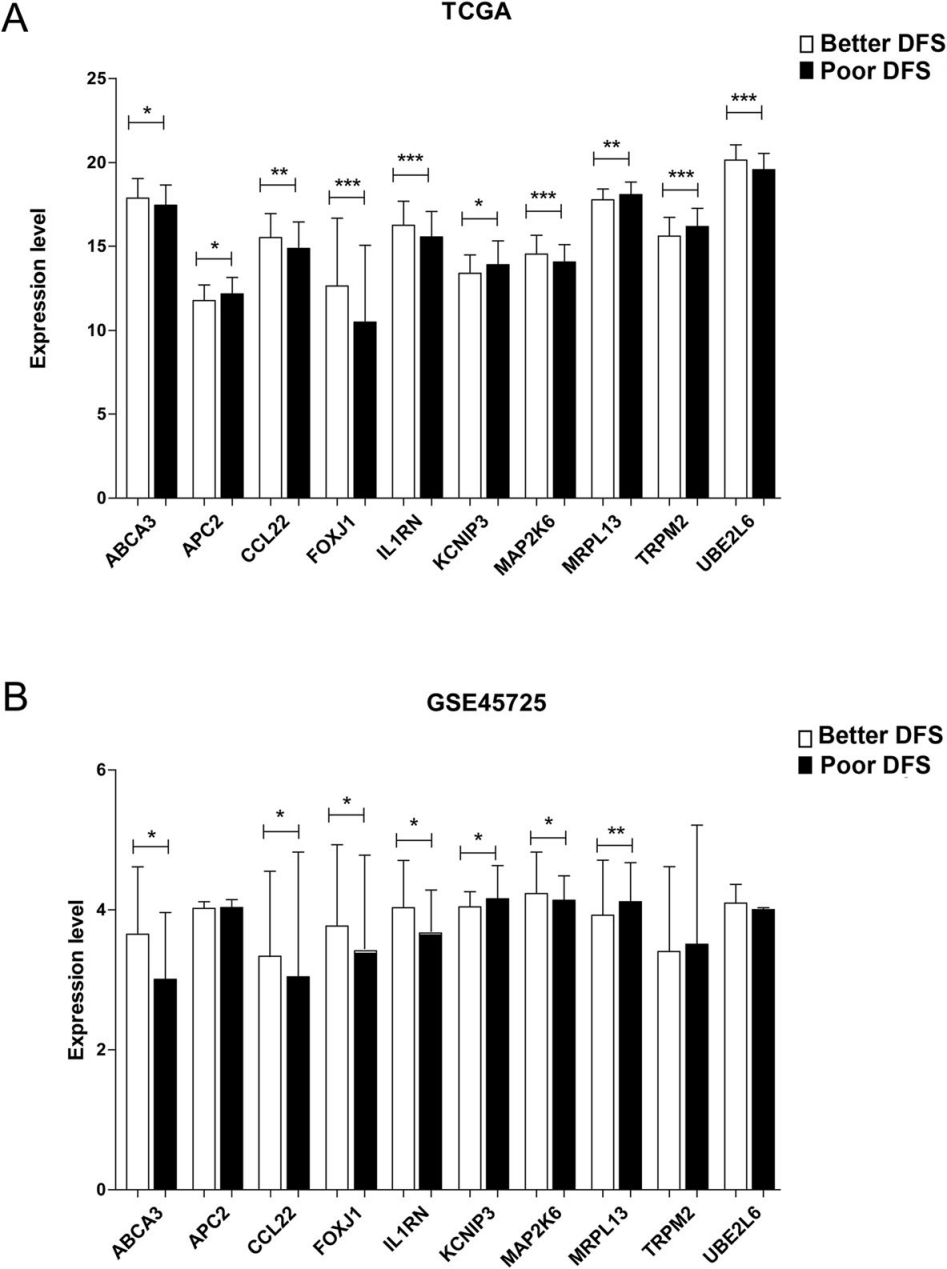
The column diagram of the expression levels of the 10 important characteristic DEGs in the TCGA training set (**a**) and the GSE45725 validation set (**b**) in the better and poor DFS groups, with white and black columns representing the better and poor DFS sample groups, respectively

The expression levels of 10 important DEGs in TCGA training set and GSE45725 validation data samples were displayed in Fig. 5. As shown in the figure, the expression level of each important DEG in the two data sets was consistent, and the 7 DEGs, *ABCA3* (*p* = 3.56E−04 in TCGA and *p* = 1.01E−02 in GSE45725), *CCL22* (*p* = 1.14E−04 in TCGA and *p* = 4.72E−02 in GSE45725), *FOXJ1* (*p* = 3.45E−05 in TCGA and *p* = 4.88E−02 in GSE45725), *IL1RN* (*p* = 5.55E−05 in TCGA and *p* = 2.19E−02 in GSE45725), *KCNIP3* (*p* = 2.68E−04 in TCGA and *p* = 2.92E−02 in GSE45725), *MAP2K6* (*p* = 5.87E−05 in TCGA and *p* = 3.28E−02 in GSE45725), and *MRPL13* (*p* = 8.27E−05 in TCGA and *p* = 9.74E−04 in GSE45725), were significantly expressed in both groups in the two data sets.

## Discussion

The mRNA expression studies have been widely used to predict the prognosis of breast cancer patients [24–26]. In this study, based on the breast cancer gene expression profile data in TCGA, 540 DEGs were screened between better DFS and poor DFS groups, including 177 downregulated and 363 upregulated DEGs. Through LR model screening, 10 important feature DEGs were identified and then validated in the GSE45725, among which, *ABCA3*, *CCL22*, *FOXJ1*, *IL1RN*, *KCNIP3*, *MAP2K6*, and *MRPL13*, were significantly expressed in both groups in the two data sets. Additionally, based on the K-M curve, it was found that *ABCA3*, *CCL22*, *FOXJ1*, *IL1RN*, and *MAP2K6* were factors associated with good prognostic outcome, while *KCNIP3* and *MRPL13* were risk factors associated with poor prognostic outcome. Our findings will improve our understanding of breast cancer and provide novel biomarkers for the prognosis and recurrence of breast cancer.

Among the five factors associated with good prognostic outcome, *CCL22*, *FOXJ1*, and *IL1RN* were found to be significantly involved in function associated with immune response. Defense against tumors is one of the functions of the immune system, and the host immune response plays a key role in progression and response to therapy of breast cancer [27]. A study has revealed that the risk of breast cancer is associated with impaired immune responses [28]. *CCL22* has an effect on repressing immune responses to tumor cells through its ability of recruiting Treg and Th2-cells, thereby enhancing tumor development [29]. Li et al. [30] has reported that *CCL22* is an independent prognostic predictor of breast cancer patients. In addition, recent studies have suggested that *FOXJ1* may be a tumor suppressor, which suppresses cell migration and invasion in ovarian cancer [31]. A study by Wang et al. [32] have demonstrated that downregulated *FOXJ1* is an independent prognostic predictor for gastric cancer, and it is found to be hypermethylated in breast tumorigenesis [33]. Our study showed that higher expression of *FOXJ1* was associated with better DFS in breast cancer, and *FOXJ1* may be a good prognostic factor for the prognosis of breast cancer. For *IL1RN*, its low expression is an early driver of carcinogenesis of urothelial carcinoma of the urinary bladder [34]. Moreover, genetic polymorphisms of *IL1RN* are found to be associated with individual susceptibility for breast cancer development in Korean women [35]. Combined with our results, it was speculated that *CCL22*, *FOXJ1*, and *IL1RN* may play an important role in ameliorating the prognosis of breast cancer patients through involving in immune response.

*ABCA3*, also a prognostic factor of a good prognostic outcome, has been reported to be involved in lipid transport and lipid secretion, and is expressed in some human epithelial cells [36]. A recent study has found that *ABCA3* has strong expression in normal mammary gland tissue and was exclusively expressed in the epithelial cell layer. The loss of ABCA3 protein expression was related to a more aggressive phenotype in breast cancer patients. The upregulation of ABCA3 was associated with a better prognosis of patients with breast cancer [37]. In accordance with the findings above, our study also suggested that upregulated *ABCA3* may be related to the good prognosis of breast cancer and served as a protective factor in breast cancer.

In addition, MAP2K6 is an upstream kinase of the p38/MAPK signaling pathway [38]. Recent studies have found that MAP2K6 may be associated with the progression of cancers [39]. *MAP2K*6 expression is found to be significantly upregulated in gastric cancer, colon cancer, and esophageal cancer compared with the control [39]. Overexpression of MAP2K6 predicts a worse prognosis of patients with nasopharyngeal carcinoma [40]. However, Wang et al. [41] reported that *MAP2K6* gene had a low expression in breast cancer compared with control. The possible reason is that the expression of *MAP2K6* in different cancers is different, and its function is complicated. In our study, we found that *MAP2K6* had a higher expression in the better DFS group compared with the poor DFS group, which indicated that high expression of *MAP2K6* may be associated with good prognosis breast cancer. Further studies are needed to investigate the specific mechanisms of *MAP2K6* in the prognosis of breast cancer.

However, *MRPL13* and *KCNIP3* were predicted to be risk factors associated with poor prognostic outcome in breast cancer. *MRPL13* was associated with the function of mitochondrial ribosome. It has been suggested that mitochondrial ribosomes are linked to tumorigenesis. The expression of genes encoding for mitochondrial ribosomal proteins is modified in numerous cancers [42, 43]. Recently, a study reported that overexpression of mitochondrial ribosomal protein S18-2 provides a permanent stimulus for cell division, which suggested its involvement in carcinogenesis [44]. The role of *MRPL13* in breast cancer has not been reported to our knowledge. For *KCNIP3*, it has been identified as a potential biomarker for the early detection of basal-like breast cancer [45]. In alignment with our results, we speculated that *MRPL13* and *KCNIP3* may be served as poor prognostic biomarkers, and their downregulation may have a better prognosis of breast cancer.

## Conclusion

In conclusion, *ABCA3*, *CCL22*, *FOXJ1*, *MAP2K6*, and *IL1RN* may serve as good prognostic factors, while *KCNIP3* and *MRPL13* may be poor prognostic biomarkers to predict the recurrence and prognosis of breast cancer. These good and poor prognostic biomarkers are required to be confirmed using larger cohorts, different validation data or different subgroups. The results will provide more powerful biomarkers for the prognosis and recurrence of breast cancer.

## Supplementary information

**Additional file 1: Table 1.** Histopathological data from TCGA database cases.

**Additional file 2: Table 2.** Histopathological data from GSE45725 dataset cases.

## Data Availability

The datasets used and analyzed in the current study are available from the corresponding author in response to reasonable requests.

## Abbreviations

DEGs: Differentially expressed genes
LR: Logit regression
FDR: False discovery rate
GO: Gene Ontology
BP: Biological process
MF: Molecular function
CC: Cellular component
KEGG: Kyoto Encyclopedia of Genes and Genomes

## References

[1] Siegel RL, Miller KD, Jemal A (2015). Cancer statistics, 2015. CA: a cancer journal for clinicians..

[2] Hendrick RE (2010). Radiation doses and cancer risks from breast imaging studies. Radiology..

[3] Zhou M, Zhong L, Xu W, Sun Y, Zhang Z, Zhao H, Yang L, Sun J (2016). Discovery of potential prognostic long non-coding RNA biomarkers for predicting the risk of tumor recurrence of breast cancer patients. Sci Rep..

[4] Mehta S, Shelling A, Muthukaruppan A, Lasham A, Blenkiron C, Laking G, Print C (2010). Predictive and prognostic molecular markers for cancer medicine. Therapeutic advances in medical oncology..

[5] Cianfrocca M, Goldstein LJ (2004). Prognostic and predictive factors in early-stage breast cancer. The oncologist..

[6] Duffy MJ, O'Donovan N, McDermott E, Crown J (2016). Validated biomarkers: the key to precision treatment in patients with breast cancer. Breast..

[7] Anothaisintawee T, Wiratkapun C, Lerdsitthichai P, Kasamesup V, Wongwaisayawan S, Srinakarin J, Hirunpat S, Woodtichartpreecha P, Boonlikit S, Teerawattananon Y (2013). Risk factors of breast cancer: a systematic review and meta-analysis. Asia Pac J Public Health..

[8] Duffy MJ, McDermott EW, Crown J (2017). Use of multiparameter tests for identifying women with early breast cancer who do not need adjuvant chemotherapy. Clin Chem.

[9] Györffy B, Lanczky A, Eklund AC, Denkert C, Budczies J, Li Q, Szallasi Z (2010). An online survival analysis tool to rapidly assess the effect of 22,277 genes on breast cancer prognosis using microarray data of 1,809 patients. Breast Cancer Res Treat..

[10] Chanrion M, Negre V, Fontaine H, Salvetat N, Bibeau F, Mac Grogan G, Mauriac L, Katsaros D, Molina F, Theillet C, Darbon JM (2008). A gene expression signature that can predict the recurrence of tamoxifen-treated primary breast cancer. Clin Cancer Res.

[11] Cronin M, Sangli C, Liu ML, Pho M, Dutta D, Nguyen A, Jeong J, Wu J, Langone KC, Watson D (2007). Analytical validation of the Oncotype DX genomic diagnostic test for recurrence prognosis and therapeutic response prediction in node-negative, estrogen receptor-positive breast cancer. Clin Chem..

[12] Knauer M, Mook S, Rutgers EJ, Bender RA, Hauptmann M, van de Vijver MJ, Koornstra RH, Bueno-de-Mesquita JM, Linn SC, van’t Veer LJ (2010). The predictive value of the 70-gene signature for adjuvant chemotherapy in early breast cancer. Breast Cancer Res Treat..

[13] Ji C, Lin S, Yao D, Li M, Chen W, Zheng S, Zhao Z (2019). Identification of promising prognostic genes for relapsed acute lymphoblastic leukemia. Blood Cells Mol Dis..

[14] Wang D-Y, Done SJ, Mc Cready DR, Leong WL (2014). Validation of the prognostic gene portfolio, ClinicoMolecular Triad Classification, using an independent prospective breast cancer cohort and external patient populations. Breast Cancer Res..

[15] Diaz-Romero J, Romeo S, Bovée JV, Hogendoorn PC, Heini PF, Mainil-Varlet P (2010). Hierarchical clustering of flow cytometry data for the study of conventional central chondrosarcoma. J Cell Physiol..

[16] Cheadle C, Vawter MP, Freed WJ, Becker KG (2003). Analysis of microarray data using Z score transformation. J Molecular Diagn..

[17] Ritchie ME, Phipson B, Wu D, Hu Y, Law CW, Shi W, Smyth GK (2015). limma powers differential expression analyses for RNA-sequencing and microarray studies. Nucleic Acids Res.

[18] Wang L, Cao C, Ma Q, Zeng Q, Wang H, Cheng Z, Zhu G, Qi J, Ma H, Nian H (2014). RNA-seq analyses of multiple meristems of soybean: novel and alternative transcripts, evolutionary and functional implications. BMC Plant Biol..

[19] Eisen MB, Spellman PT, Brown PO, Botstein D (1998). Cluster analysis and display of genome-wide expression patterns. Proc Natl Acad Sci..

[20] Tikole S, Sankararamakrishnan R (2006). A survey of mRNA sequences with a non-AUG start codon in RefSeq database. J Biomol Struct Dyn..

[21] Sherman BT, Lempicki RA (2009). Systematic and integrative analysis of large gene lists using DAVID bioinformatics resources. Nat Protoc..

[22] Huang DW, Sherman BT, Lempicki RA (2008). Bioinformatics enrichment tools: paths toward the comprehensive functional analysis of large gene lists. Nucleic Acids Res..

[23] Wang P, Wang Y, Hang B, Zou X, Mao J-H (2016). A novel gene expression-based prognostic scoring system to predict survival in gastric cancer. Oncotarget..

[24] Sorlie T, Tibshirani R, Parker J, Hastie T, Marron J, Nobel A, Deng S, Johnsen H, Pesich R, Geisler S (2003). Repeated observation of breast tumor subtypes in independent gene expression data sets. Proc Natl Acad Sci U S A..

[25] Van De Vijver MJ, He YD, Van't Veer LJ, Dai H, Hart AA, Voskuil DW, Schreiber GJ, Peterse JL, Roberts C, Marton MJ (2002). A gene-expression signature as a predictor of survival in breast cancer. New England J Med..

[26] Reis-Filho JS, Pusztai L (2011). Gene expression profiling in breast cancer: classification, prognostication, and prediction. Lancet..

[27] Thompson E, Taube JM, Elwood H, Sharma R, Meeker A, Warzecha HN, Argani P, Cimino-Mathews A, Emens LA (2016). The immune microenvironment of breast ductal carcinoma in situ. Mod Pathol..

[28] Na-Jin P, Kang D-H (2006). Breast cancer risk and immune responses in healthy women. Oncol Nurs Forum.

[29] Nishikawa H, Sakaguchi S (2010). Regulatory T cells in tumor immunity. Int J Cancer..

[30] Li Y-Q, Liu F-F, Zhang X-M, Guo X-J, Ren M-J, Fu L (2013). Tumor secretion of CCL22 activates intratumoral Treg infiltration and is independent prognostic predictor of breast cancer. Plos One..

[31] Siu MK, Wong ES, Kong DS, Chan HY, Jiang L, Wong OG, Lam EW, Chan KK, Ngan HY, Le X-F (2013). Stem cell transcription factor NANOG controls cell migration and invasion via dysregulation of E-cadherin and FoxJ1 and contributes to adverse clinical outcome in ovarian cancers. Oncogene..

[32] Wang J, Cai X, Xia L, Zhou J, Xin J, Liu M, Shang X, Liu J, Li X, Chen Z (2015). Decreased expression of FOXJ1 is a potential prognostic predictor for progression and poor survival of gastric cancer. Ann Surg Oncol..

[33] Demircan B, Dyer LM, Gerace M, Lobenhofer EK, Robertson KD, Brown KD (2009). Comparative epigenomics of human and mouse mammary tumors. Genes Chromosomes Cancer..

[34] Worst TS, Reiner V, Gabriel U, Weiß C, Bolenz C (2014). IL1RN and KRT13 Expression in bladder cancer: association with pathologic characteristics and smoking status. Adv Urol..

[35] Lee K-M, Park SK, Hamajima N, Tajima K, Choi J-Y, Noh D-Y, Ahn S-H, Yoo K-Y, Hirvonen A, Kang D (2006). Genetic polymorphisms of interleukin-1 beta (IL-1B) and IL-1 receptor antagonist (IL-1RN) and breast cancer risk in Korean women. Breast cancer research and treatment..

[36] Stahlman MT, Besnard V, Wert SE, Weaver TE, Dingle S, Xu Y, Kv Z, Olson SJ, Whitsett JA (2007). Expression of ABCA3 in developing lung and other tissues. J Histochem Cytochem..

[37] Schimanski S, Wild P, Treeck O, Horn F, Sigruener A, Rudolph C, Blaszyk H, Klinkhammer-Schalke M, Ortmann O, Hartmann A (2010). Expression of the lipid transporters ABCA3 and ABCA1 is diminished in human breast cancer tissue. Horm Metab Res..

[38] Cuenda A, Lizcano JM, Lozano J (2017). Mitogen activated protein kinases. Frontiers Cell Dev Biol..

[39] Parray AA, Baba RA, Bhat HF, Wani L, Mokhdomi TA, Mushtaq U, Bhat SS, Kirmani D, Kuchay S, Wani MM (2014). MKK6 is upregulated in human esophageal, stomach, and colon cancers. Cancer Invest..

[40] Li Z, Li N, Shen L (2018). MAP2K6 is associated with radiation resistance and adverse prognosis for locally advanced nasopharyngeal carcinoma patients. Cancer Manag Res..

[41] Wang H-J, Zhou M, Jia L, Sun J, Shi H-B, Liu S-L, Wang Z-Z (2015). Identification of aberrant chromosomal regions in human breast cancer using gene expression data and related gene information. Med Sci Monit..

[42] Gogvadze V, Orrenius S, Zhivotovsky B (2008). Mitochondria in cancer cells: what is so special about them?. Trends Cell Biol..

[43] Koc EC, Haciosmanoglu E, Claudio PP, Wolf A, Califano L, Friscia M, Cortese A, Koc H (2015). Impaired mitochondrial protein synthesis in head and neck squamous cell carcinoma. Mitochondrion..

[44] Mints M, Mushtaq M, Iurchenko N, Kovalevska L, Stip MC, Budnikova D, Andersson S, Polischuk L, Buchynska L, Kashuba E (2016). Mitochondrial ribosomal protein S18-2 is highly expressed in endometrial cancers along with free E2F1. Oncotarget..

[45] Labaer J, Wang J, Qiu J, Wallstrom G, Anderson K, Park J, Figueroa J (2017). Plasma autoantibody biomarkers for basal like breast cancer.

