# Correction to: Prognostic biomarkers related to breast cancer recurrence identified based on Logit model analysis

**DOI:** 10.1186/s12957-020-02079-0

**Published:** 2020-12-05

**Authors:** Xiaoying Zhou, Chuanguang Xiao, Tong Han, Shusheng Qiu, Meng Wang, Jun Chu, Weike Sun, Liang Li, Lili Lin

**Affiliations:** 1 Department of Nursing, Wuxi Higher Health Vocational Technology School, Wuxi, 2140128 Jiangsu China; 2 Department of Breast Thyroid Surgery, Central Hospital of Zibo, Zibo, 255036 Shandong China; 3 Department of Rehabilitation Medicine, Xinxiang Medical University, Xinxiang, 453000 Henan China; 4 Department of Pharmacy, Wuxi Higher Health Vocational Technology School, No. 305, Xinguang Road, Wuxi, 214028 Jiangsu China

**Correction to: World J Surg Onc 18, 254 (2020)**

**https://doi.org/10.1186/s12957-020-02026-z**

Following publication of the original article [1], the authors identified an error in the affiliation of Meng Wang and Jun Chu. Their institution should be “2 Department of breast thyroid surgery, Central Hospital of Zibo, Zibo, 255036, Shandong, China”.

The correct affiliation have been included in this correction. The authors apologize for this error.
